# Supplementary material for: OncoGEMINI: software for investigating tumor variants from multiple biopsies with integrated cancer annotations
Source: Genome Med. 2021 Mar 26;13:46. doi: 10.1186/s13073-021-00854-6 (PMC7995589; doi:10.1186/s13073-021-00854-6)
Supplement: Supplementary file 1 — Additional file 1. : Supplementary Methods for the running of FreeBayes. [file 13073_2021_854_MOESM1_ESM.docx]

**Additional Files**

**Supplementary Methods**

FreeBayes was used to create individual VCFs corresponding to SNVs and INDEL variants for each patient described in the metastatic breast cancer spatial analysis. BAM files for each sample were downloaded from the ENA and used by FreeBayes version 1.3.1 with the following parameters:

--pooled-continuous

--pooled-discrete

--genotype-qualities

--report-genotype-likelihood-max

--allele-balance-priors-off

--min-alternate-fraction 0.03

--min-repeat-entropy 1

--min-alternate-count 2

These parameters were selected to be intentionally lenient and enable sensitive variant calling in the exome data.
